# Renal dysfunction and prognosis of COVID-19 patients: a hospital-based retrospective cohort study

**DOI:** 10.1186/s12879-021-05861-x

**Published:** 2021-02-08

**Authors:** Hui-Xian Xiang, Jun Fei, Ying Xiang, Zheng Xu, Ling Zheng, Xiu-Yong Li, Lin Fu, Hui Zhao

**Affiliations:** 1 Second Affiliated Hospital, Anhui Medical University, Furong Road no 678, Hefei, 230601 Anhui Province China; 2 Second People’s Hospital of Fuyang City, Fuyang, 236015 Anhui Province China; 3 Department of Toxicology, Anhui Medical University, 81 Meishan Road, Hefei, 230032 Anhui Province China

**Keywords:** Severe acute respiratory syndrome coronavirus-2, Coronavirus disease 2019, Renal dysfunction, Prognosis

## Abstract

**Introduction:**

Increasing evidence indicate that coronavirus disease 2019 (COVID-19) is companied by renal dysfunction. However, the association of Severe Acute Respiratory Syndrome Coronavirus-2 (SARS-CoV-2)-induced renal dysfunction with prognosis remains obscure.

**Materials and methods:**

All 154 patients with COVID-19 were recruited from the Second People’s Hospital of Fuyang City in Anhui, China. Demographic characteristics and laboratory data were extracted. Renal dysfunction was evaluated and its prognosis was followed up based on a retrospective cohort study.

**Results:**

There were 125 (81.2%) mild and 29 (18.8%) severe cases in 154 COVID-19 patients. On admission, 16 (10.4%) subjects were accompanied with renal dysfunction. Serum creatinine and cystatin C were increased and estimated glomerular filtration rate (eGFR) was decreased in severe patients compared with those in mild patients. Renal dysfunction was more prevalent in severe patients. Using multivariate logistic regression, we found that male gender, older age and hypertension were three importantly independent risk factors for renal dysfunction in COVID-19 patients. Follow-up study found that at least one renal function marker of 3.33% patients remained abnormal in 2 weeks after discharge.

**Conclusion:**

Male elderly COVID-19 patients with hypertension elevates the risk of renal dysfunction. SARS-CoV-2-induced renal dysfunction are not fully recovered in 2 weeks after discharge.

## Background

At the winter of 2019, Coronavirus Disease 2019 (COVID-19), an emerging infectious disease with unclear etiology broke out in Wuhan City, Hubei Province, China [1]. Later, this unknown virus was clarified and named as severe acute respiratory syndrome coronaviruse-2 (SARS-CoV-2) [2, 3]. Now, it has been pandemic across the world. Up to January 1st, 2021, there were approximate 90 million accumulated confirmed patients of SARS-CoV-2 infections in 220 countries, of them about one million cases have died [4]. All humanity is sustained huge disaster from SARS-CoV-2 [5, 6]. Therefore, all medical staff, scientific research personnel and all sectors of the entire society must cooperate to decrease COVID-19 spread and seek for effective cures drugs and method.

Previous studies have demonstrated that COVID-19 patients mainly accompanied with fever, diarrhea, dry cough, lymphocyte reduction and radiographic evidence of pneumonia [7]. Now, more and more studies have confirmed that SARS-CoV-2 not only evoked severe acute respiratory syndrome, but also induced multiple organ injuries, such as myocardial injury, lymphocyte reduction and even liver dysfunction [8–11]. Nevertheless, the clinical characteristics of renal dysfunction caused by SARS-CoV-2 are rarely described. Moreover, the clinical significance of SARS-CoV-2-induced renal dysfunction and its recovery situation are still ambiguous.

This research primarily explored whether SARS-CoV-2-evoked renal dysfunction, its risk factors and prognosis after discharge among COVID-19 patients. Our data reveal that male, higher age and hypertension are three independently risk factors of renal dysfunction. Our research firstly demonstrates that SARS-CoV-2-evoked renal dysfunction are not fully recovered in 2 weeks after discharge.

## Materials and methods

### Subjects

In this study, 154 COVID-19 patients were enrolled from the Second People’s Hospital of Fuyang City of Anhui Province from January 1 to February 30, 2020. The Second People’s Hospital of Fuyang City was the designated infectious hospital in Fuyang City. All patients were diagnosed with SARS-CoV-2 injection using RT-PCR on pharyngeal swab specimens. The diagnostic criteria of SARS-CoV-2 injection was referred to the New Coronavirus Pneumonia Prevention and Control Program (8th edition). There was no death in Fuyang City. The severity of patients with COVID-19 were accessed through oxygenation index based on the New Coronavirus Pneumonia Prevention and Control Program [12]. Mild case, defined as oxygenation index higher than 300; For severe case, whose oxygenation index was from 200 to 300; For critically ill case, whose oxygenation index was lower than 200. Finally, the prognosis of patients with COVID-19 was observed in 2 weeks after discharge among 150 subjects. Renal function was assessed in 150 patients with COVID-19. This project was approved by the institutional ethics board of the Second People’s Hospital of Fuyang City (No. 2020-5). Individual signed informed consent was gained from patients.

### Data collection

Demographic data and clinical characteristics were collected. The severity of COVID-19 was evaluated. The hospital stay was calculated in COVID-19 patients. Uric acid, urea nitrogen, creatinine, cystatin C and estimated glomerular filtration rate (eGFR) were measured. All laboratory tests were performed in the clinical laboratory of the Second People’s Hospital of Fuyang City.

### Statistical analysis

Statistical analyses were conducted using SPSS software (version 19.0). Categorical variables were expressed with frequencies and percentages. All continuous variables were shown as medians with interquartile ranges (P25, P75). Categorical variables were evaluated using Fisher exact test or χ^2^ test. Continuous variables were analyzed through ANOVA and Mann-Whitney U test. Moreover, the main risk factors of renal dysfunction were examined using multivariate logistic regression models and potential confounders were adjusted. Obvious differences were reported with *P* values less than 0.05.

## Results

### Demographic information and clinical manifestations

All 154 COVID-19 patients were enrolled and analyzed in the Second People’s Hospital of Fuyang City of Anhui Province. We found that mild patient, was 125 (81.2%) (Table 1). Severe patient accounted for 18.8% (Table 1). Besides, the demographic data were evaluated. As shown in Table 2, of COVID-19 patients, 92 (59.7%) were male and 62 (40.3%) were female. There were 69 patients <39 years old, 68 patients aged between 40 and 59 years, and 17 patients >60 years old. Among 154 COVID-19 patients, 22 (14.3%) subjects accompanied with hypertension, 8 (5.19%) with diabetes and 12 (7.79%) subjects with other chronic diseases.

**Table 1:** The association between the severity of COVID-19 patients and renal functional indexes

Parameters	Mild	Severe
Cases, N (%)	125 (81.2)	29 (18.8)
Uric acid (μmol/L)	239.0 (180.0, 306.8)	218.0 (186.0, 247.5)
Urea nitrogen (mmol/L)	3.7 (3.1, 4.7)	4.0 (2.8, 5.4)
Creatinine (μmol/L)	65.0 (52.0, 77.0)	70.0 (54.5, 83.5) *
Cystatin C (mg/L)	0.81 (0.70, 0.92)	0.89 (0.71, 0.93) *
eGFR (mL/min)	126.4 (103.4, 150.6)	118.6 (99.3, 142.7) *
Renal dysfunction, N (%)	5 (4.0)	11 (37.9)

The levels of renal function were expressed with median (P25, P75)

Renal dysfunction was defined as any of renal functional indexes beyond normal range

*P< 0.05

**Table 2:** The effects of demographic characteristics and complications on renal function indexes

	Cases, N (%)	Uric acid (μmol/L)	Urea nitrogen (mmol/L)	Creatinine (μmol/L)	Cystatin C (mg/L)	eGFR (mL/min)
**Gender**						
Male	92 (59.7)	274.0 (218.0, 336.0)	4.3 (3.6, 5.2)	75.0 (66.0, 82.0)	0.84 (0.75, 0.94)	110.3 (97.0, 133.1)
Female	62 (40.3)	201.0 (157.5, 245.0) **	3.2 (2.7, 3.6) *	51.5 (44.0, 57.0) **	0.72 (0.64, 0.81) *	142.2 (124.3, 175.3) **
**Age**						
<39	69 (44.8)	253.0 (191.5, 306.5)	3.6 (3.2, 4.3)	65.0 (49.5, 78.5)	0.76 (0.66, 0.87)	133.5 (108.5, 181.6)
40–59	68 (44.2)	243.0 (186.0, 329.0)	3.6 (3.0, 4.8)	63.5 (53.0, 76.3)	0.80 (0.71, 0.94)	122.9 (103.4, 142.9) *
>60	17 (11.0)	288.0 (229.5, 347.0) *^##^	5.3 (3.6, 6.9) *^#^	72.0 (60.5, 87.0) *^#^	0.90 (0.81, 1.39) **^#^	105.4 (84.5, 121.1) ^##^
**Hypertension**						
Yes	22 (14.3)	225.0 (195.0, 354.5)	5.1 (3.1, 6.7)	65.0 (52.0, 78.0)	0.89 (0.75, 1.07)	110.0 (94.3, 144.1)
No	132 (85.7)	235.5 (178.3, 295.5)	3.7 (3.1, 4.6)**	66.0 (52.0, 77.0)	0.80 (0.69, 0.89) *	126.4 (103.2, 148.7)
**Diabetes**						
Yes	8 (5.2)	327.0 (235.0, 372.0)	6.3 (5.5, 10.6)	72.0 (57.0, 108.0)	0.99 (0.87, 1.54)	106.2 (63.9, 133.0)
No	146 (94.8)	234.0 (182.0, 298.0) **	3.7 (3.1, 4.6)**	65.0 (52.0, 77.0) *	0.80 (0.70, 0.90) **	126.0 (103.3, 150.1) *
**Other diseases**						
Yes	12 (7.8)	227.0 (190.5, 344.5)	4.5 (2.5, 6.0)	69.0 (59.5, 76.8)	0.90 (0.81, 0.94)	109.8 (96.0, 129.8)
No	142 (92.2)	236.0 (181.0, 298.0)	3.7 (3.1, 4.7)	65.0 (52.0, 77.8)	0.79 (0.70, 0.91)	126.5 (103.3, 150.1) *

The levels of renal function were expressed with median (P25, P75)

Cases in Gender, compared with “Male”, *P< 0.05, **P< 0.01

Cases in Age, compared with “< 39”, *P< 0.05, **P< 0.01; Compared with “40–59”, ^#^*P*< 0.05, ^##^*P*< 0.01

Cases in Hypertension, Diabetes and Other diseases, compared with “Yes”, *P< 0.05, **P< 0.01

### Correlation of renal dysfunction and the severity in COVID-19 patients

**Fig. 1:**
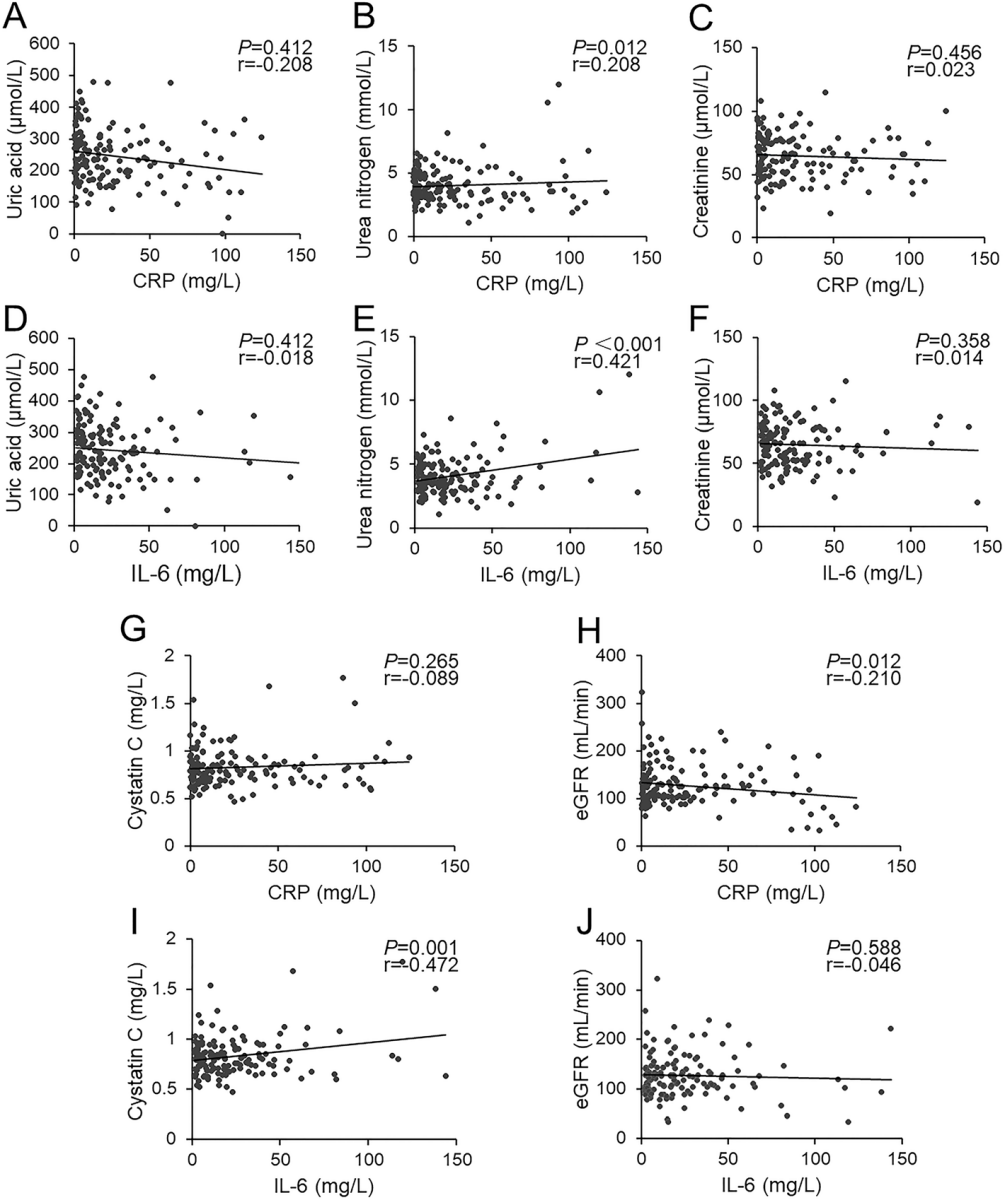
The correlations between inflammatory cytokines and renal function indexes among COVID-19 patients. **a, d** Correlations between inflammatory cytokines and uric acid were analyzed among COVID-19 patients. **a** CRP; (D) IL-6. **b, e** Correlations between inflammatory cytokines and urea nitrogen were analyzed among COVID-19 patients. **b** CRP; **e** IL-6. **c, f** Correlations between inflammatory cytokines and creatinine were analyzed among all COVID-19 patients. **c** CRP; **f** IL-6. **g, i** Correlations between inflammatory cytokines and cystatin C were analyzed among all COVID-19 patients. **g** CRP; **i** IL-6. **h, j** Correlations between inflammatory cytokines and cystatin C were analyzed among all COVID-19 patients. **h** CRP; **j** IL-6

The correlation between renal dysfunction and the severity of COVID-19 was analyzed. Serum renal function indexes, including uric acid, urea nitrogen, creatinine, cystatin C and eGFR, were measured. As shown in Table 1, the level of serum creatinine and cystatin C were higher, the level of serum eGFR was lower in severe patients than those in mild patients. There was no difference of uric acid, urea nitrogen and cystatin C between mild patients and severe patients. Renal dysfunction was defined as any of renal functional indexes beyond normal range. Our results indicated that 5 (4.0%) cases with renal dysfunction were in mild patients and 11 (37.9%) cases with renal dysfunction were in severe patients on admission. Besides, the correlations between renal function indexes and inflammatory cytokines were analyzed. As shown in Fig. 1, no significant correlations were observed between inflammatory cytokines with uric acid and creatinine. Moreover, there was a weekly positive correlation between urea nitrogen with CRP (*r*=0.208, *P*=0.012) and IL-6 (*r*=0.421, *P*<0.001). Further analysis indicated that cystatin C was negatively correlated with IL-6 (*r*=− 0.472, *P*=0.001) and eGFR was inversely correlated with CRP (*r*=− 0.210, *P*=0.012) among COVID-19 patients.

### Male elderly cases with hypertension elevated the risk of renal dysfunction

The influences of demographic characteristics and complications on renal dysfunction were accessed among COVID-19 patients. As shown in Table 2, the level of uric acid, urea nitrogen, creatinine and cystatin C were higher and eGFR were lower in males than those in females. The effect of age on renal function was analyzed. The results revealed that the level of eGFR was lower in patients between 40 to 59 years than those of younger than 39 years. Moreover, our results also revealed that the levels of uric acid, urea nitrogen, creatinine and cystatin C were higher in patients >60 years than those younger patients. The level of eGFR was lowest in the patients >60 years. In addition, the effects of comorbidities on renal dysfunction were then accessed. As shown in Table 2, urea nitrogen was increased and cystatin C was reduced in COVID-19 patients with hypertension. In addition, uric acid, urea nitrogen, creatinine and cystatin C were increased and eGFR was decreased in COVID-19 patients with diabetes. Meanwhile, we found that the level of eGFR was slightly lower in COVID-19 patients with other chronic diseases than those without chronic disease. In addition, the risk factors of renal dysfunction were analyzed using multivariate logistic regression among COVID-19 patients. As shown in Table 3, the *OR* of male gender was 1.465 (*95% Cl*: 1.124, 4.001), the *OR* of age was 1.945 (*95% Cl*: 1.024, 3.128) and the *OR* of hypertension was 2.345 (*95% Cl*: 1.135, 3.458) for renal dysfunction, respectively. Diabetes and other diseases had no obvious effect on renal dysfunction in COVID-19 patients (Table 3).

**Table 3:** Multivariable logistic regression analysis between demographics characteristics and complications with renal dysfunction among COVID-19 patients

	β	Wald	*P* value	OR (95% CI)
Male	0.584	4.123	0.008	1.465 (1.124, 4.001)
Age	0.784	6.512	0.021	1.945 (1.024, 3.128)
Hypertension	0.457	2.354	0.031	2.345 (1.135, 3.458)
Diabetes	0.468	2.324	0.564	0.865 (0.654, 1.231)
Other diseases	0.384	1.128	0.387	0.721 (0.324, 1.178)

### Renal function indexes were abnormal in 2 weeks after discharge

The recovery condition of renal dysfunction was investigated in COVID-19 patients. Renal function indexes were measured and compared between on admission and in 2 weeks after discharge. As shown in Table 4, there was no significant difference of urea nitrogen, creatinine, cystatin C and eGFR between on admission and after discharge in COVID-19 patients. However, the level of uric acid was increased in 2 weeks after discharge than on admission. On admission, 4 (2.6%) patients with uric acid, 4 (2.6%) patients with urea nitrogen, 1 (0.6%) patient with creatinine and 6 (3.9%) patients with cystatin C were above the normal range. Besides, 6 (3.9%) patients with eGFR were below the normal range. There was total 16 (10.4%) COVID-19 subjects with renal dysfunction on admission. The prognosis of COVID-19 patients with renal dysfunction was tracked until 2 weeks after discharge. After discharge, 1 (0.7%) patient with creatinine and 2 (1.4%) patients with cystatin C were above the normal range. Additionally, 2 (1.4%) patients with eGFR were below the normal range (Table 4). Further analysis found that 5 (3.33%) COVID-19 patients in total still remained with renal dysfunction in 2 weeks after discharge (Table 4).

**Table 4:** Renal functional indexes on admission and after discharge among COVID-19 patients

Renal indexes	On admission (*N*=154)			Discharge (*N*=150)		
	Median (P25, P75)	Below the range, N (%)	Above the range, N (%)	Median (P25, P75)	Below the range, N (%)	Above the range, N (%)
Uric acid (μmol/L)	235.0 (183.0, 299.5)	52 (33.8)	4 (2.6)	283.0 (237.0, 351.0) *	43 (28.6)	0^#^
Urea nitrogen (mmol/L)	3.7 (3.1, 4.7)	35 (22.7)	4 (2.6)	3.9 (3.4, 4.8)	17 (11.5)	0^#^
Creatinine (μmol/L)	65.5 (52.0, 77.0)	3 (1.9)	1 (0.6)	59.0 (48.0, 71.0)	5 (3.4)	1 (0.7)
Cystatin C (mg/L)	0.8 (0.7, 0.9)	15 (9.7)	6 (3.9)	0.76 (0.69, 0.87)	14 (9.5)	2 (1.4)
eGFR (mL/min)	125.8 (103.0, 148.6)	6 (3.9)	86 (55.8)	134.1 (118.7, 156.5)	2 (1.4)	72 (48.6)
Renal dysfunction, N (%)		16 (10.4%)			5 (3.33%)^†^	

Compared with “Median values” among COVID-19 patients on admission

Compared with “Above the range” among COVID-19 patients on admission, ^#^*P*< 0.05

Compared with “On admission”, ^†^*P*< 0.05

*P<0.05

## Discussion

This research primarily analyzed SARS-CoV-2-evoked renal dysfunction, its risk factors and prognosis in 2 weeks after discharge in a hospital-based retrospective cohort study. Our results indicate that male gender, older age and hypertension are three independently risk factors of renal dysfunction among COVID-19 patients. In addition, SARS-CoV-2-evoked renal dysfunction was not fully recovered in 2 weeks after discharge.

Mounting evidences have confirmed that SARS-CoV-2 injection evoked multiple organ injuries, primarily containing liver dysfunction, myocardial injury, lymphocyte reduction and even respiratory failure [8–11]. In this research, the levels of serum uric acid, urea nitrogen, creatinine, cystatin C and eGFR were detected and renal dysfunction was evaluated among COVID-19 patients between on admission and discharge. Our results indicated that creatinine and cystatin C were increased, eGFR was decreased in severe COVID-19 patients. No difference of uric acid, urea nitrogen and cystatin C were observed between mild and severe COVID-19 patients. Furthermore, the number of COVID-19 patients with renal dysfunction was more in severe patients than those in mild patients. Renal dysfunction was more pervasive in severe patients with COVID-19. Our data indicate that renal dysfunction is positively correlated with the severity of COVID-19 patients on admission.

Earlier studies have confirmed that older age elevated the risk of death in COVID-19 patients [12, 13]. Several comorbidities aggravated the severity of COVID-19 patients [14, 15]. In this research, the influences of demographic characteristics on renal dysfunction were evaluated. No difference of urea nitrogen was observed in different gender patients. Uric acid and cystatin C were decreased, eGFR was increased in females. Additionally, we found that the levels of uric acid, urea nitrogen, creatinine and cystatin C were higher and the level of eGFR was lower in the older patients. Furthermore, the influences of basic complications on renal dysfunction were analyzed. Our results indicated that the levels of urea nitrogen and cystatin C were increased in COVID-19 patients with hypertension. Further analysis found that uric acid, urea nitrogen, creatinine and cystatin C were increased and eGFR was decreased in COVID-19 patients with diabetes. Furthermore, we also found that eGFR was decreased in COVID-19 patients with other chronic diseases. These evidences suggest that male gender, older age, diabetes and hypertension may aggravate the risk of renal dysfunction in COVID-19 patients. For the sake of analyzing the risk factors of renal dysfunction among COVID-19 patients, multivariate logistic regression analysis was carried out. These data demonstrate that male gender, older age and hypertension are three independent risk factors of renal dysfunction among COVID-19 patients. In brief, male elderly subjects with hypertension elevates the risk of renal dysfunction in COVID-19 patients.

The prognosis of renal dysfunction remained obscure among COVID-19 patients. This is an important doubt which is worthy of exploring whether renal dysfunction of COVID-19 patients is reversed in 2 weeks after discharge. In the present project, 150 cases with COVID-19 were followed up and renal dysfunction were accessed. Every index of renal function and the rate of renal dysfunction were compared between on admission and in 2 weeks after discharge among COVID-19 patients. No difference of urea nitrogen, creatinine, cystatin C and eGFR were confirmed between on admission and after discharge. However, the level of uric acid was slightly increased in 2 weeks after discharge compared with on admission among COVID-19 patients. Moreover, the abnormal number of patients with creatinine, cystatin C and eGFR were similar between on admission and in 2 weeks after discharge. Interestingly, a few patients with serum creatinine, cystatin C and eGFR were still out of the normal range in 2 weeks after discharge. These data suggest that renal function indexes of 3.33% cases with COVID-19 were not fully recovered in 2 weeks after discharge. Hence, whether SARS-CoV-2-caused a long-time renal dysfunction is required to explore in the next therapeutic methods.

The mechanism of SARS-CoV-2-caused renal dysfunction is still unknown. More and more reports have revealed that SARS-CoV-2 plays a pathogenetic role in COVID-19 patients through binding to receptor of angiotension converting enzyme (ACE)2 [16, 17]. At present, several researches demonstrated that ACE2 can express in renal tubular epithelium [18]. Therefore, we speculate that SARS-CoV-2 may directly damage kidney tissue through binding to the ACE2 receptor. Besides, earlier studies found that inflammatory cytokines were dramatically elevated in patients with COVID-19 [19–21]. It is generally known that cytokine storm was associated with the process and the levels of IL-6 and CRP can predict the severity and prognosis of COVID-19 patients [22, 23]. IL-6 is a multifunctional cytokine that transmits cell signaling and regulates immune cells [24]. CRP is an acute-phase proinflammatory cytokine and a sensitive biomarker of infection and tissue damage [25]. The secreting of CRP or IL-6 always induces cytokine storm and damages multiple organs function. In this study, the correlations between inflammatory cytokines and renal dysfunction were evaluated among COVID-19 patients. The results indicated that there were positive correlations between renal dysfunction with IL-6 and CRP in COVID-19 patients. Consequently, these results reveal that cytokine storm may involve in the process of SARS-CoV-2-induced renal dysfunction.

There are several weaknesses in this study. Firstly, this was only a single center research, all patients were from Fuyang City in Anhui Province. Source of patients may cause selection bias. All patients with COVID-19 were timely found and treated in the Fuyang City, so COVID-19 patients only were mild and severe cases in Fuyang City. The prevalence was very low and severity of renal dysfunction was modest. Secondly, renal dysfunction was evaluated only through the linear determination of biomarkers and not in an accepted and comparable way (AKIN, KADIG, etc), more assessment methods are needed to evaluate the renal dysfunction in the next project. Thirdly, because of minor specimen, a larger sample size is needed to perform. Fourthly, this current study was only a hospital-based retrospective cohort study, the mechanism by which SARS-CoV-2-induced renal dysfunction in COVID-19 patients was unclear. We can’t exclude the effect of drug use patterns and doses on renal dysfunction in COVID-19 patients. More animal studies and in vivo experiment are needed to conduct in the future research.

## Conclusion

All in all, this project primarily described SARS-CoV-2-evoked renal dysfunction in the Second People’s Hospital of Fuyang City of Anhui Province. These data reveal that renal dysfunction is more prevalent in severe patients with COVID-19. Moreover, male gender, older age and hypertension are significant differently risk factors for enal dysfunction. Our results demonstrate that renal dysfunction is not fully restored in 2 weeks after discharge in COVID-19 patients. Hence, whether SARS-CoV-2 evokes a long-period renal dysfunction in COVID-19 patients is required to evaluated in COVID-19 patients.

## Data Availability

The data generated during and/or analyzed during the current study are available from the corresponding author on reasonable request.

## Abbreviations

COVID-19: Coronavirus disease 2019
SARS-CoV-2: Severe Acute Respiratory Syndrome Coronavirus-2
IL-6: Interleukin-6
CRP: C-reactive protein
ACE: Angiotension converting enzyme
eGFR: Estimated glomerular filtration rate
